# A185 ERYTHEMA NODOSUM AS A MARKER FOR OBJECTIVE DISEASE ACTIVITY IN INFLAMMATORY BOWEL DISEASE: A SINGLE CENTER RETROSPECTIVE STUDY

**DOI:** 10.1093/jcag/gwae059.185

**Published:** 2025-02-10

**Authors:** S Sasson, I Kalisky, G Rosenfeld, B Bressler

**Affiliations:** University of British Columbia, Department of Medicine, Vancouver, BC, Canada; The IBD Centre of British Columbia, Division of Gastroenterology, Vancouver, BC, Canada; The IBD Centre of British Columbia, Division of Gastroenterology, Vancouver, BC, Canada; The IBD Centre of British Columbia, Division of Gastroenterology, Vancouver, BC, Canada

## Abstract

**Background:**

Erythema Nodosum (EN) is an inflammatory condition characterized by tender, erythematous nodules, typically found on the extensor surfaces of the extremities. EN is linked to both immune-mediated conditions, such as inflammatory bowel disease (IBD), Behçet’s syndrome, and sarcoidosis, as well as non-immune-mediated conditions. In patients with IBD, EN affects 3–10% of those with ulcerative colitis (UC) and 4–15% with Crohn’s disease (CD), particularly in patients with colonic involvement. While EN has been thought to be associated with disease activity, this conclusion has been primarily based on symptom assessment, and a definitive association with objective measures, such as inflammatory markers or endoscopic activity, has not yet been established.

**Aims:**

To investigate the association of EN to objective inflammatory assessments among IBD patients.

**Methods:**

Using the IBD Center of British Columbia database, patients with active EN were assessed. For each patient, disease activity based on symptoms was evaluated. Objective assessments, such as colonoscopy and imaging modalities such as CT, MR, or video capsule enterography, were included if they were performed within a three-month window of the EN presentation. Fecal calprotectin and C-reactive protein (CRP) were collected if obtained within four weeks of the EN presentation, using thresholds of 250 µg/g and 5 mg/L respectively.

Standard descriptive analyses were conducted to evaluate any association between both objective and subjective measures of disease activity and the presence of EN.

**Results:**

The initial search identified 165 patients with a documented presentation of EN. The mean age at presentation of EN was 40 years. The majority of EN patients had Crohn’s disease (89, 76%), and were predominantly female (86, 74%). Among those, 116 patients had at least one objective assessment within the prespecified time with 71 patients (61%) having a colonoscopy as a sole method to identify disease activity, while CRP, imaging, and fecal calprotectin were used in 28 (24%), 10 (9%), and 7 (6%) of the cases, respectively.

In regards to disease activity, 68 (58%) patients had gastrointestinal symptoms at the time of EN presentation. However, out of 116 cases, a total of 93 (80%) had objective evidence of active disease.

**Conclusions:**

In this analysis of IBD patients with active EN, objective evidence of intestinal disease activity was found in a significant majority (80%) of cases, despite only 58% of patients reporting gastrointestinal symptoms at the time of EN presentation. These findings suggest that EN may be closely associated with underlying intestinal inflammation even in the absence of overt clinical symptoms. This highlights the potential importance of pursuing objective assessments in patients who present with EN, even with no overt clinical symptoms.

**Funding Agencies:**

None

